# Modelling traffic-induced multicomponent ultrafine particles in urban street canyon compartments: Factors that inhibit mixing^☆^

**DOI:** 10.1016/j.envpol.2018.03.002

**Published:** 2018-07

**Authors:** Jian Zhong, Irina Nikolova, Xiaoming Cai, A. Rob MacKenzie, Roy M. Harrison

**Affiliations:** a School of Geography, Earth & Environmental Sciences, University of Birmingham, Edgbaston, Birmingham, B15 2TT, UK; b Birmingham Institute of Forest Research, University of Birmingham, Edgbaston, Birmingham, B15 2TT, UK; c Department of Environmental Sciences/Center of Excellence in Environmental Studies, King Abdulaziz University, PO Box 80203, Jeddah, 21589, Saudi Arabia

**Keywords:** Urban aerosol, Canyon structure, Box model, Aerosol microphysics, Canyon compartmentalisation

## Abstract

This study implements a two-box model coupled with ultrafine particle (UFP) multicomponent microphysics for a compartmentalised street canyon. Canyon compartmentalisation can be described parsimoniously by three parameters relating to the features of the canyon and the atmospheric state outside the canyon, i.e. the heterogeneity coefficient, the vortex-to-vortex exchange velocity, and the box height ratio. The quasi-steady solutions for the two compartments represent a balance among emissions, microphysical aerosol dynamics (i.e. evaporation/condensation of semi-volatiles, SVOCs), and exchange processes, none of which is negligible. This coupled two-box model can capture significant contrasts in UFP number concentrations and a measure of the volatility of the multi-SVOC-particles in the lower and upper canyon. Modelled ground-level UFP number concentrations vary across nucleation, Aitken, and accumulation particle modes as well-defined monotonic functions of canyon compartmentalisation parameters. Compared with the two-box model, a classic one-box model (without canyon compartmentalisation) leads to underestimation of UFP number concentrations by several tens of percent typically. By quantifying the effects of canyon compartmentalisation, this study provides a framework for understanding how canyon geometry and the presence of street trees, street furniture, and architectural features interact with the large-scale atmospheric flow to determine ground-level pollutant concentrations.

## Highlights

• Ultrafine particle multicomponent microphysics is coupled with a 2-box canyon model.
• Significant contrasts in particle number concentrations in the canyon are captured.
• Particle number concentrations vary across modes as functions of canyon parameters.
• A one-box model would underestimate ground-level particle number concentrations.

## Graphical abstract

**Figure:**
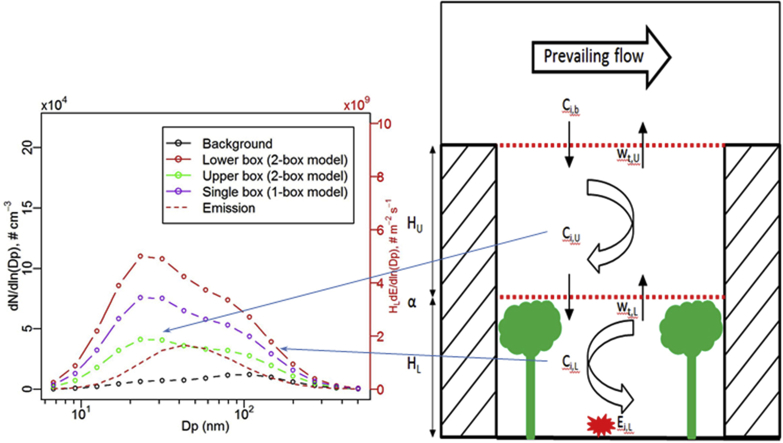

## Teaser

Effects of canyon compartmentalisation on ultrafine particle (UFP) number concentrations and volatility are revealed by the two-box model coupled with UFP multicomponent microphysics.

## 1 Introduction

Urban air pollution induced by road traffic is a key environmental concern (Murena et al., 2009). As one of the major urban pollutants, particulate matter (PM) has received much attention in the scientific community (Dall'Osto et al., 2011; Heal et al., 2012). PM_10_ (with an aerodynamic diameter d_p_ < 10 μm) and PM_2.5_ (d_p_ < 2.5 μm) are currently regulated in terms of the mass concentrations of particles (US EPA, 2017b; European Commission, 2017). Although regulations for ultrafine particle (UFP or PM_0.1_, d_p_ < 0.1 μm) do not yet exist, UFP is a very significant contribution to total particle number concentrations (Harrison et al., 2000). UFP may accumulate in the lungs (Panis et al., 2010) or penetrate cells/tissue (Geiser et al., 2005), causing health effects because of their small sizes. Semi-volatile components of UFP may also contribute to secondary organic aerosol formation (Baldauf et al., 2016).

An urban street canyon is a linear urban feature having buildings on both sides of a street (Li et al., 2008). In such an environment, ground-level atmospheric flow is restricted by the buildings, which may lead to reduced air ventilation between the street canyon and the overlying atmospheric background (Salim et al., 2011). According to the canyon aspect ratio (*AR*, the ratio of building height *H* to street width *W*), street canyons may be categorized into deep (*AR*≥2), regular (0 < *AR*<2), and avenue (*AR*≤0.5) (Vardoulakis et al., 2003). Deep street canyons present worst-case scenarios for the dispersion of air pollutants (Li et al., 2009), since there may be multiple segregated vortices formed in the canyon, which can lead to even poorer ventilation conditions. Below, we call such segregated inhibition of mixing within the street canyon, *compartmentalisation*. The presence of street trees, street furniture, and architectural features can also lead to compartmentalisation in shallower street canyons and may create multiple split vortices with reduced exchange. Street trees (Gromke et al., 2008) or architectural elements, e.g. roof shapes (Takano and Moonen, 2013), balconies (Murena and Mele, 2016) and elevated expressways (Huang and Zhou, 2013), may produce an internal ‘lid’ that constrains the height of the primary street vortex (Gromke and Ruck, 2007).

The microphysical and/or chemical processes associated with mixing across compartments, together with emissions and the exchange with background air can be parsimoniously represented by a two-box model. The concept of a two-box model was previously introduced and evaluated against field measurements (Murena et al., 2011; Murena, 2012) to predict carbon monoxide (CO) concentration (taken as a passive scalar because of its long chemical lifetime) in a deep street canyon and no chemical processes were considered. The traditional one-box model (originally assuming a single vortex in a regular canyon) may not be appropriate for deep street canyon scenarios (with canyon compartmentalisation) (Murena et al., 2011; Murena, 2012). Zhong et al. (2015) adopted simple NO_*x*_-O_3_ (nitrogen oxides-ozone) photochemistry into a two-box model (representing two segregated vortices found in their large eddy simulation LES of a deep canyon with AR = 2) and there was a good agreement between the LES model and the two-box model. Zhong et al. (2017) further coupled more complex O_3_-NO_*x*_-VOC (nitrogen oxides-ozone-volatile organic compounds) chemistry into both LES and a two-box model for a deep street canyon. Concentrations of oxidants were found to be increased by about 30–40% via the additional OH/HO_2_ (hydroxyl/hydroperoxyl radicals) chemistry compared with simple NO_*x*_-O_3_ photochemistry adopted in Zhong et al. (2015). The pre-processing within the canyon could enhance oxidant fluxes from the canyon to the overlying atmospheric background, with an even greater effect for deep street canyons than shallower street canyons. Zhong et al. (2016) employed the two-box model coupled with O_3_-NO_*x*_-VOC chemistry to investigate effects of governing parameters (i.e. heterogeneity coefficient, exchange velocity and box height ratio) for a variety of emission scenarios and to identify under which conditions NO_2_ (nitrogen dioxide) at the pedestrian level would exceed its air quality limit value.

The current study extends the two-box modelling approach by including the multicomponent microphysics of UFP in urban street canyon compartments. The canyon-box modelling approach is similar conceptually to that of Pugh et al. (2012b) but has been coded independently. The UFP code for the present study is shared with that of CiTTy-Street-UFP (Nikolova et al., 2016), i.e. the CiTTyCAT (Pugh et al., 2012a) model coupled with UFP microphysics.

## 2 Methods

### 2.1 Framework of a two-box model coupled with UFP

**Fig. 1:**
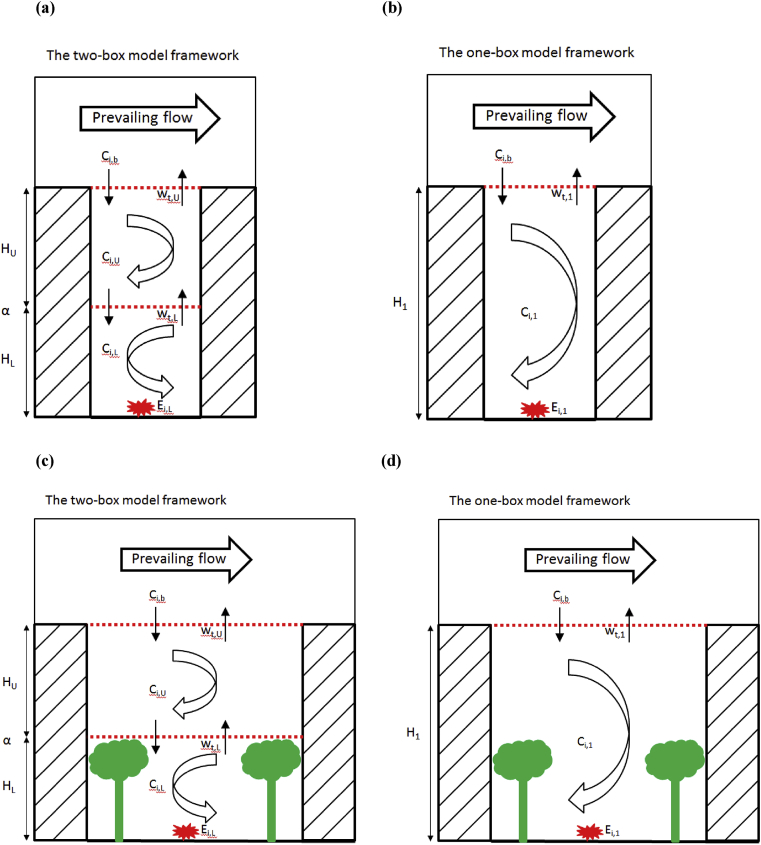
Framework of the coupled two-box and one-box models: examples of a deep, smooth-walled canyon (a–b) and a regular canyon with tree canopy interrupting/restricting the circulation at the lower canyon (c–d). “C” and “*i*” denotes the concentration of *i*th tracer in both particulate and gas phases (Equations (1), (2), (3), (4), (15), (16)).

The two-box model based on vortex structure from the LES model for a deep street canyon (AR = 2) was previously implemented for both simple NO_*x*_-O_3_ and more complex O_3_-NO_*x*_-VOC chemistry, and evaluated against the LES-chemistry models (Zhong et al., 2015, 2016, 2017). The extension of this simplified two-box model to the multicomponent microphysics of UFP concerning emissions, microphysical aerosol dynamics (i.e. evaporation/condensation of semi-volatiles, SVOCs), and exchange processes in a compartmentalised street canyon (Fig. 1) for both particulate and gas phases is described below. For the particulate phase:

(1) $$\frac{dQ_{q,j,U}}{dt}=\frac{w_{t,L}}{H_{U}}\left(N_{j,L}-N_{j,U}\right)\chi_{q,j,L}m_{j}-\frac{w_{t,U}}{H_{U}}\left(N_{j,U}-N_{j,b}\right)\chi_{q,j,U}m_{j}+\Delta Q_{q,j,U}$$

(2) $$\frac{dQ_{q,j,L}}{dt}=-\frac{w_{t,L}}{H_{L}}\left(N_{j,L}-N_{j,U}\right)\chi_{q,j,L}m_{j}+E_{q,j,L}+\Delta Q_{q,j,L}$$

where “_*q*_” represents the component *q*; “*j*” is the size bin *j*; “L” and “U” represent the lower and upper boxes, respectively; “_*b*_” represents the overlying background; “*Q*” denotes the mass concentration in the particulate phase; “*N*” is the number concentration; “χ” is the mass fraction; “*m*” is the mass of one representative particle in a sectional bin; “*w*_*t*_” is the exchange velocity (the exchange/diffusion process are based on the number concentration gradient); “*H*” is the height of the box; “*E*” is the emission rate into the lower box volume per unit time; ΔQ denotes the source terms for the particulate phase from the UFP module due to aerosol transformation processes (e.g. condensation/evaporation in this study).

For the gas phase,

(3) $$\frac{dc_{q,U}}{dt}=\frac{w_{t,L}}{H_{U}}\left(c_{q,L}-c_{i,U}\right)-\frac{w_{t,U}}{H_{U}}\left(c_{q,U}-c_{q,b}\right)+\Delta c_{q,U}$$

(4) $$\frac{dc_{q,L}}{dt}=-\frac{w_{t,L}}{H_{L}}\left(c_{q,L}-c_{q,U}\right)+E_{q,L}+\Delta c_{q,L}$$

where *c* is the mass concentration in the gas phase; Δc denotes the source terms for the gas phase from the UFP module due to aerosol transformation processes; other symbols are same as those in Equations (1), (2)). In this study, the source terms (Equations (1), (2), (3), (4))) are derived from the UFP module due to particle condensation/evaporation (further details in Section 2.2), rather than from the chemistry module in previous studies (Zhong et al., 2015, 2016, 2017). The number of UFP components used in the model is 18: 1 non-volatile core and 17 surrogate Semi-Volatile Organic Compounds (SVOC) (parameterised as n-alkanes from C_16_H_34_ to C_32_H_66_) (Nikolova et al., 2016). The present model runs use 15 sectional size bins, ranging from 6.7 nm to 501.4 nm in a uniform logarithmic scale. The UFP number concentration in a size bin is calculated based on the total mass concentrations in a size bin (divided by the dry aerosol mass per particle in the given size bin). There are 17 tracers in the gas-phase corresponding to each SVOC component. Sequential ordinary differential equations in the model are solved on a 0.3 s time step for emission/exchange processes and adaptive time steps for aerosol evaporation/condensation processes.

For deep canyons (AR ≥ 2), the spontaneous formation of primary and secondary vortices motivates the use of multiple boxes (Fig. 1a and b); for other values of AR, street trees, street furniture, and architectural features may all lead to zones of inhibited mixing (Fig. 1c and d) that motivate a multi-box approach (Gromke and Ruck, 2007; Huang and Zhou, 2013; Gromke et al., 2008).

### 2.2 Condensation/evaporation of semi-volatiles

The condensation/evaporation process of semi-volatiles (SVOCs) is one of the most important aerosol transformation processes in predicting the fate of ultrafine particles in urban air (Harrison et al., 2016). This process is driven by the difference between the partial pressure of a gas species and its saturation vapour pressure over a particle surface (Jacobson, 2005), which will alter the size of the particle. The condensation/evaporation rate of each component (*q*) of SVOCs, i.e. for n-alkanes from C_16_H_34_ to C_32_H_66_, in a size bin *j* is estimated based on the mass flux between the gas phase and particles, i.e.

(5) $$\frac{dm_{q,j}}{dt}=a_{FS}^{q,j}\frac{2\pi d_{j}M_{q}D_{q}}{RT}\left(e_{q}^{\infty }-X_{q,j}a_{K}^{q,j}e_{q}^{vap}\right)$$

where *d*_*j*_ is the particle diameter (m); *M*_*q*_ is the molar mass of SVOC component *q* (g mol^−1^), and *X*_*q,j*_ is the mole fraction of component *q* in size bin *j*; *D*_*q*_ is the vapour diffusivity of component *q* (m^2^ s^−1^); R is the universal gas constant (J mol^−1^ K^−1^); T is temperature (K); $e_{q}^{\infty }$ is the ambient partial pressure of component *q* (Pa), which can be calculated from gas concentrations of SVOCs via the ideal gas law. The initial gas conditions for SVOCs are specified as a urban background site (Harrad et al., 2003) (Table S1); $e_{q}^{vap}$ is the saturation vapour pressure (Pa) of SVOC component *q* over a pure, flat, surface, and is estimated at a temperature of 278.15 K representing a winter scenario (Table S1) based on EPI suite v4.1 (US EPA, 2017a) (widely used for the estimation of saturation vapour pressures in the literature (Harrison et al., 2016; Nikolova et al., 2016; Sangiorgi et al., 2014; Shin et al., 2014; Wei et al., 2016); $a_{K}^{q,j}$ is the Kelvin effect term of SVOC component *q* in size bin *j*:

(6) $$a_{K}^{q,j}=\exp\left(\frac{4\delta v_{q}}{RTd_{j}}\right)$$

with δ the particle surface tension (N m^−1^) and $v_{q}$ the molar volume of SVOC component *q* (m^3^ mol^−1^); $a_{FS}^{q,j}$ is the Fuchs-Sutugin correction factor for non-continuous effects:

(7) $$a_{FS}^{q,j}=\frac{1+Kn_{j}}{1+\left(\frac{4}{3A_{q}}+0.377\right)Kn_{j}+\frac{4}{3A_{q}}Kn_{j}^{2}}$$

with $A_{q}$ the accommodation coefficient for SVOC component *q* on the particle surface – a value of 1 is specified for each component (Julin et al., 2014) and $Kn_{j}$ the Knudsen number applicable to the gas condensing onto, or evaporating from, particles in size bin *j*:

(8) $$Kn_{j}=\frac{2\lambda }{d_{j}}$$

λ is the mean free path of the air (m). A positive value of $\frac{dm_{q,j}}{dt}$ (kg s^−1^) represents a condensation process (i.e., a positive particle velocity along the particle-size axis) while a negative value represents an evaporation process for a SVOC component *q* at size bin j.

The source term of each component *q* in the gas phase (Equations (3), (4))) due to the condensation and evaporation processes can be derived:

(9) $$\Delta c_{q,L}=-\sum_{j}\frac{N_{j}dm_{q,j}}{dt}$$

For the particulate phase, the particle would change to a new size (*d*_*n*_) due to the condensation and evaporation processes and a redistribution scheme is required to redistribute particle number and mass concentration onto the sectional size bin. It is assumed that *d*_*n*_ falls between two adjacent sectional size bin, *j* and *j*+1.

(10) $$N_{j}=\frac{d_{j+1}^{3}-d_{n}^{3}}{d_{j+1}^{3}-d_{j}^{3}}N_{n}$$

(11) $$N_{j+1}=\frac{d_{n}^{3}-d_{j}^{3}}{d_{j+1}^{3}-d_{j}^{3}}N_{n}$$

(12) $$Q_{q,j}=\frac{d_{j+1}^{3}-d_{n}^{3}}{d_{j+1}^{3}-d_{j}^{3}}N_{n}\chi_{q,n}m_{j}$$

(13) $$Q_{q,j+1}=\frac{d_{n}^{3}-d_{j}^{3}}{d_{j+1}^{3}-d_{j}^{3}}N_{n}\chi_{q,n}m_{j+1}$$

The source term for the particular phase (Equations (1), (2))) due to the condensation and evaporation processes can be then diagnosed after the redistribution on a sectional size bin.

Here, we use a simple particle composition metric to show the impact of compartmentalisation on UFP composition, i.e. the mass-fraction-weighted SVOC carbon number (MFWCN):

(14) $$\bar{C_{j}}=\sum_{q=1}^{17}\chi_{q,j,SVOC}C_{q}$$

where *χ*_*q,j,SVOC*_ is the mass fraction of the SVOC component (excluding non-volatile core) with carbon number, *C*_q_, in size bin j.

### 2.3 Canyon compartmentalisation

For the purpose of assessing the effect of canyon compartmentalisation, the one-box model (assuming a “well-mixed” box for the whole street canyon without compartmentalisation) is also configured and can be described below. For the particulate phase:

(15) $$\frac{dQ_{q,j,1}}{dt}=-\frac{w_{t,1}}{H_{1}}\left(N_{j,1}-N_{i,b}\right)\chi_{q,j,1}m_{j}+E_{q,j,1}+\Delta Q_{q,j,1}$$

For the gas phase:

(16) $$\frac{dc_{q,1}}{dt}=-\frac{w_{t,1}}{H_{1}}\left(c_{q,1}-c_{q,1}\right)+E_{q,1}+\Delta S_{q,1}$$

Similarly, the symbols are represented by “1” while “U” and “L” are used in the two-box model (Equations (1), (2), (3), (4))). Deposition of pollutants to hard or vegetated surfaces within the (one- or two-box) street canyon is not considered; this has been shown elsewhere to be a significant component of the pollutant mass balance when retention times are high (Pugh et al., 2012b). Using the SVOC scheme discussed here, and a single-box approach, Nikolova et al. (2016) calculated UFP number concentrations at steady-state, and found reductions of ∼4% and ∼5% in total UFP number for low wind speed conditions due to deposition and coagulation, respectively.

It is assumed that $N_{i,L}$ from the more realistic two-box model is the “true” value of UFP number concentration and there would be an error if the well-mixed “one-box” model is used to predict the UFP number concentration in the lower canyon, i.e.

(17) $$\Delta N_{j,L}=N_{j,1}-N_{j,L}$$

Further, the percentage of underestimation by the “one-box” model (compared with the more “realistic” two-box model) to predict the number concentration in the lower part of the compartmentalised canyon (where human exposure takes place) can be defined as follows:

(18) $$\varphi_{j,L}=\frac{\Delta N_{j,L}}{N_{j,L}}\times 100\%$$

Canyon compartmentalisation can be described parsimoniously by three parameters, i.e. the heterogeneity coefficient (*η*), the exchange velocity $\left(w_{t,1}\right)$, and the box height ratio (*α*) (See Equations S1-S7 in Supporting Information for details), representing the influence of the key features of street canyon and the key drivers of wind/turbulence. Zhong et al. (2016) defined the heterogeneity coefficient to represent the spatial variability across the two boxes, i.e. $\eta =1-\frac{c_{ps,U}}{c_{ps,1}}=1-\frac{w_{t,1}}{w_{t,U}}$ (S5) ranging from 0 to 1. A value of η=0 represents two homogenous (well-mixed) boxes, and a higher value of *η* means the concentration difference between the two boxes would become higher. An increased value of *η* can be interpreted as a reduced exchange between the lower and upper canyon, which may be associated with less vehicle-induced turbulence, fewer roughness elements, or the presence of a dense tree canopy (Pugh et al., 2012b; Vos et al., 2013). The exchange velocity in the one-box model, $w_{t,1}$, is defined based on a steady state of the street canyon system with a passive scalar emission and is dependent more on the large scale meteorological conditions. An increased value of *w*_t,1_ can be interpreted as a higher wind speed above the canyon or a higher turbulent intensity near the roof-top level induced, e.g., by roof-top geometries. The box height ratio is defined as the ratio of the lower box height to the whole canyon height, i.e. $\alpha =H_{L}/H_{1}$ (S3). The box height ratio will be determined by the street canyon geometry as well as the flow structure emerging from the interaction with the above-canyon flow. An increased value of *α* can be interpreted as a larger vortex below capped by a smaller vortex above, which may be formed in a pitched-roof scenario, or when the canyon-bottom air is driven by thermals due to solar radiation or other heating sources. The exchange velocities to be used in the two-box model (Fig. 1) can be then derived as $w_{t,U}=\frac{w_{t,1}}{1-\eta }$ (S6) and $w_{t,L}=\frac{\alpha w_{t,1}}{\eta }$ (S7).

The two-box model requires input parameters (such as heterogeneity coefficient; exchange velocities; box height ratio; initial/background/emitted gas and particle composition) and generates time-dependent gas and particle-number concentrations in the upper and lower boxes, along with UFP size distributions and size-dependent particle compositions. The microphysical parameters (including initial gas concentration and compositional saturation vapour pressures shown in Table S1) in the UFP module are detailed in Supporting Information. In what follows, we focus on model results for scenarios with varied heterogeneity coefficient, exchange velocity, and box height ratio, in order to investigate the interplay between UFP microphysics and in-canyon mixing.

### 2.4 Model scenarios

An overview of case settings to represent the key parameters in the two-box model is given in Table 1. In the “BASE” Case, η= 0.5, $w_{t,1}=0.02\;$ m s^−1^ and α = 0.5 are set; η= 0.5 reflects a median level of heterogeneity (Murena et al., 2008); $w_{t,1}=0.02\;$ m s^−1^ represents a low wind speed above the street canyon (∼2 m s^−1^) and may be derived based on street canyon large-eddy simulations (Zhong et al., 2015; Bright et al., 2013); α = 0.5 means that the two boxes have the same volume. Emissions of UFPs are assumed to be released into the lower box only. The emission size distribution of UFPs used in this study is a bi-modal log-normal distribution with peaks at 35 nm and 65 nm (previously used by Nikolova et al. (2016)), which have a mass fraction of 1% and 90% non-volatile core, respectively; The fractional composition of SVOC in the particles (i.e. n-alkanes from C_16_H_34_ to C_32_H_66_) is then scaled based on the measured emission data in a road tunnel (He et al., 2008) (a confined space and less influenced by atmosphere conditions, which may be used for the specification of traffic emission). The particle number emission factor is based on Jones and Harrison (2006). The background UFP size distribution has a major peak at 100 nm with a minor peak at 25 nm, i.e. similar to the curve-fit profile for BT tower in London (Dall'Osto et al., 2011), representing an overlying urban background above street canyons which is less influenced by local emissions. In order to investigate the effect of one parameter, cases with changes of this parameter are configured while keeping other parameters same as those in Case BASE. As the lower canyon is the primary place of interest for human exposure, this study will mainly focus on the number concentration size distribution of UFPs in the lower canyon box for different scenarios.

**Table 1:** Overview of the model scenarios.

Case	Heterogeneity coefficient (η)	Exchange velocity *w*_t,1_ (m s^−1^)	Exchange velocity *w*_t,L_ (m s^−1^)	Exchange velocity *w*_t,U_ (m s^−1^)	Box height ratio (α)
BASE	0.5	0.02	0.02	0.04	0.5
*η*-LL	0.1	0.02	0.10	0.022	0.5
*η*-L	0.3	0.02	0.033	0.029	0.5
*η*-H	0.7	0.02	0.014	0.067	0.5
*η*-HH	0.9	0.02	0.011	0.20	0.5
*w*_t,1_-LL	0.5	0.012	0.012	0.024	0.5
*w*_t,1_-L	0.5	0.016	0.016	0.032	0.5
*w*_t,1_-H	0.5	0.024	0.024	0.048	0.5
*w*_t,1_-HH	0.5	0.028	0.028	0.056	0.5
α-LL	0.5	0.02	0.004	0.04	0.2
α-L	0.5	0.02	0.012	0.04	0.35
α-H	0.5	0.02	0.028	0.04	0.65
α-HH	0.5	0.02	0.036	0.04	0.8

Note: ‘BASE’ is the base case. ‘*η*’ denotes the heterogeneity coefficient; ‘*w*_t,1_’ denotes the exchange velocity in the one-box model; ‘α’ denotes the box height ratio. ‘LL’, ‘L’, ‘H’ and ‘HH’ represent an even lower, lower, higher and even higher value than the corresponding component in the case BASE, respectively. *w*_t,L_ and *w*_t,U_ are derived based on Equations S6 and S7 for the input parameters in the two-box model.

## 3 Results and discussion

### 3.1 BASE case number concentration size distribution

**Fig. 2:**
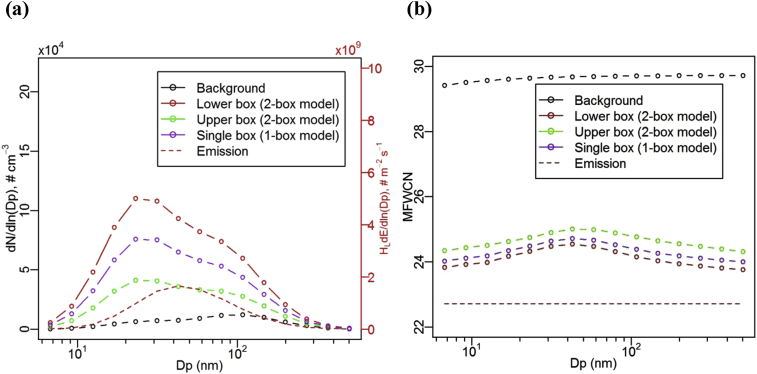
(a) Number concentration size distribution of UFP and (b) mass-fraction-weighted SVOC carbon number (MFWCN, Equation (14)), at the quasi-steady state by the 2-box model and the 1-box model for Case BASE.

Fig. 2 (a) illustrates the number concentration size distribution (NCSD) of UFPs simulated by the two-box model and the one-box model for Case BASE at a quasi-steady state (Zhong et al., 2016), here at output time, t = 240 min (a characteristic time scale for street canyon exchange may be estimated by *H*_1_/*w*_t1_). The two-box model can capture a significant contrast in NCSD between the lower and upper boxes. The NCSD in the lower box (where UFP emissions are released) is more influenced by emissions, with much higher levels than those in the upper box (which is more influenced by the overlying background atmosphere). This is consistent with the findings for photo-chemically reactive gas species (Zhong et al., 2015, 2016, 2017). The one-box model predicts a single NCSD between those in the lower and upper box from the two-box model. There is also clear evidence of evaporation of UFP for all distributions and the shrinkage of peak diameter (centred at bin 5 with bin bounds of [19.8 nm, 26.9 nm]) compared with the UFP emission profile (centred at bin 7 with bin bounds of [36.6 nm, 49.9 nm]), which may be due to the rapid evaporation especially caused by the lower carbon-number with much higher saturation vapour pressures (Table S1). The decrease in particle size due to evaporation of UFP was also indicated by field observations (Harrison et al., 2016).

The quasi-steady NCSD in the model street canyon environment is a balance between emissions, aerosol dynamics (e.g. evaporation/condensation of semi-volatiles), and exchange processes, none of which is negligible. The results from the one-box model tend to give underestimations compared with the two-box model in terms of the lower box concentration. In a deep or poorly mixed compartmentalised canyon environment, the single box assumption is not appropriate and a more “realistic”, but still computationally tractable, two-box assumption more plausibly represents the situation. The underestimations by the one-box model are quantified in following sections for different scenarios.

Fig. 2 (b) further illustrates the mass-fraction-weighted SVOC carbon number (MFWCN, defined in Equation (14)) for case BASE. MFWCN represents particle SVOC composition as a single real number related to the carbon number of each surrogate n-alkane in the particle. Since saturation vapour pressures decrease exponentially with increasing carbon number (Table S1), a higher MFWCN means a less volatile particle (as a whole), and vice versa. Therefore, it is reasonable to use MFWCN as an indicator of an “aggregated volatility” of a multi-SVOC-particle. Detailed multicomponent mass concentration size distributions for case BASE are shown in Fig. S1, where mass concentrations of SVOCs are relatively lower for smaller size bins (due to strong evaporation).

In the model, size-dependent composition evolves from the size-independent compositions of the emissions and background (Fig. 2b), as aerosol particles of a given size relax towards different quasi-steady MFWCNs in each canyon compartment. Particle evaporation is important in determining the quasi-steady MFWCNs; they are not produced by a simple blending of the emissions and background MFWCNs. The one-box model has MFWCNs between those for the lower box and the upper box from the two-box model, but somewhat nearer that of the lower box, indicating a non-linearity in the dynamical balance among emissions, microphysical aerosol dynamics (i.e. evaporation/condensation of SVOCs), and exchange processes.

### 3.2 Effect of heterogeneity coefficient

**Fig. 3:**
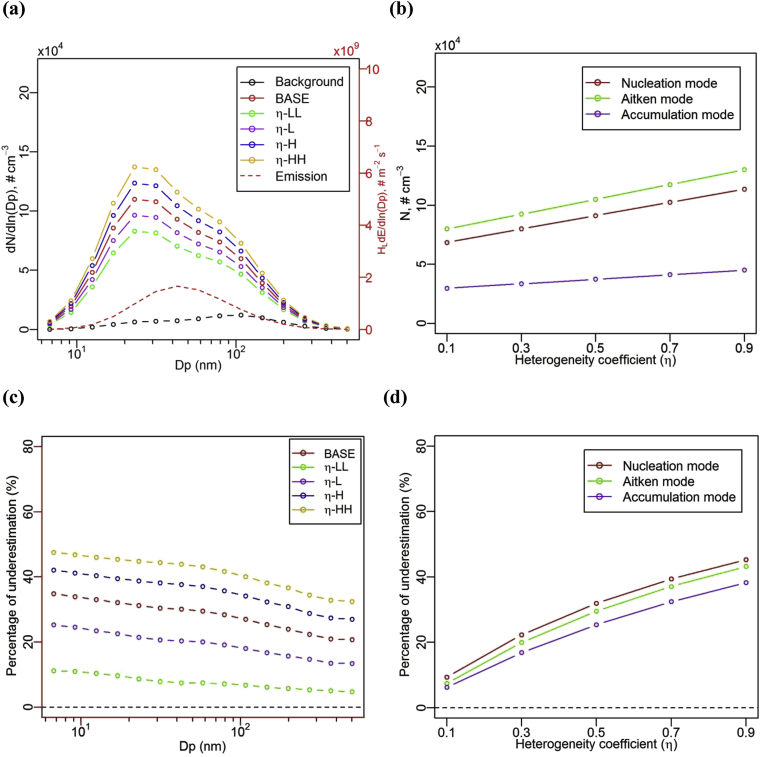
Effect of heterogeneity coefficient (η) on (a) number concentration size distribution of UFP, (b) number concentrations of UFP for different modes, (c) the percentage of underestimation by the ‘one-box’ model in different size and (d) in different modes, for the lower canyon at the quasi-steady state.

Fig. 3 (a) illustrates the effect of heterogeneity coefficient (η) on the NCSD of UFP in the lower canyon at a quasi-steady state, i.e. for Case *η*-LL (η=0.1), Case *η*-L (η=0.3), Case BASE (η=0.5), Case *η*-H (η=0.7) and Case *η*-HH (η=0.9). These profiles have similar patterns with peak diameters in bin 5 ([19.8 nm, 26.9 nm]). As expected, the number concentrations in the lower canyon increase with the increase in heterogeneity coefficient. This lower-box enhancement is less significant for both small particles (sub-10 nm with very fast evaporation due to the Kelvin effect) and very large particles (diameters above 200 nm, with more limited capacity to change size by evaporation due to Kelvin effect). Lower heterogeneity coefficients may be attributed to more local traffic-induced turbulence in the lower canyon (Murena et al., 2011) and less dense tree canopy (Gromke and Ruck, 2012), which would increase the ventilation between the lower and upper canyon boxes and thereby increasing the removal rate of particles from the pedestrian level to higher altitudes of the street canyon. Fig. 3 (b) illustrates the effect of heterogeneity coefficient on number concentrations of UFP for different modes, i.e. integrating the number size distribution in Fig. 3 (a) over nucleation mode (dp: <30 nm), Aitken mode (dp: 30–100 nm) and accumulation mode (dp: >100 nm), in the lower canyon at quasi-steady state. In general, the number concentrations of UFP at those modes increase with the increase in the heterogeneity coefficient, also indicated by Fig. 3 (a). The number concentrations of UFP have the lowest values and slopes (as a function of the heterogeneity coefficient) in the accumulation mode, followed by the nucleation mode and then the Aitken mode. This new finding of different slopes per mode suggests that the variation of NCSD with different values of *η* (associated with in-canyon ventilation) is not constant with particle size. The shallowest slope, for the accumulation mode, is possibly due to the relatively lower UFP emissions in that mode. The nucleation mode has relatively higher slope, partially attributed to the additional source of particles into this mode from the evaporation of particles in the Aitken mode. The highest slope, in the Aitken mode, is attributed to the fact that the peak diameter of UFP emissions is in the Aitken mode.

Fig. 3 (c) illustrates the percentage of underestimation by the ‘one-box’ model on the NCSD in the lower canyon under a variety of heterogeneity coefficients (η), compared to the NCSD from the two-box model. The underestimation in the number concentration in the lower canyon increases with the heterogeneity coefficient (e.g. as local traffic-induced turbulence decreases or tree canopy density increases) and is higher for smaller size bins (where evaporation is more dominant than larger size bins). The underestimation could reach up to about 48% for bin 1 in Case HC-HH and can be as low as about 5% for bin 15 in Case HC-LL. Fig. 3 (d) further shows that the percentage of underestimation by the ‘one-box’ model is more pronounced in nucleation mode than other modes (Aitken mode followed by accumulation mode). This may indicate that the diameter shrinkage (from other modes to nucleation mode) due to the evaporation would increase the error due to the single well-mixed box assumption.

### 3.3 Effect of exchange velocity

**Fig. 4:**
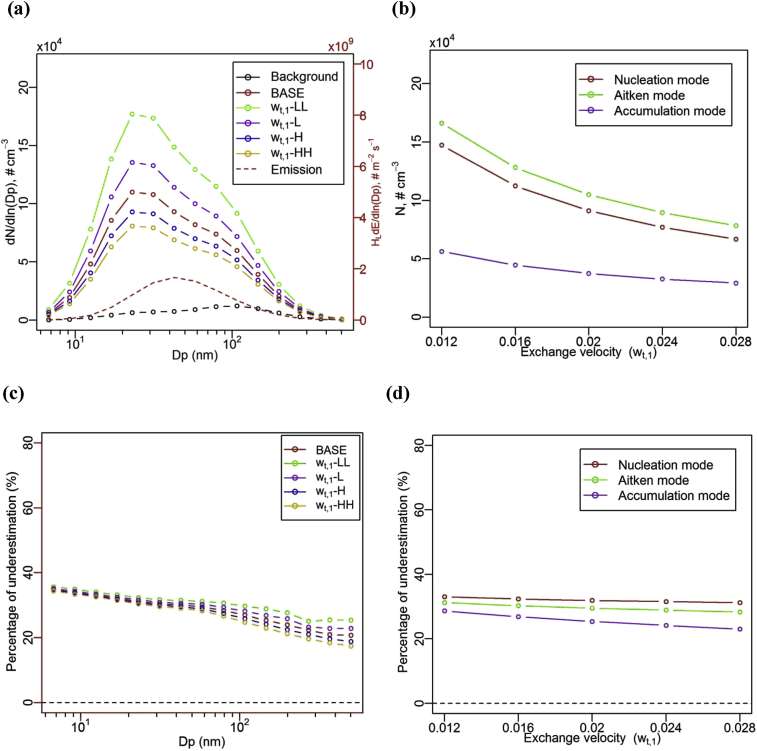
Effect of exchange velocity (*w*_t,1_) on (a) number concentration size distribution of UFP, (b) number concentrations of UFP for different modes, (c) the percentage of underestimation by the ‘one-box’ model in different size and (d) in different modes, for the lower canyon at the quasi-steady state.

Fig. 4 (a) illustrates the effect of exchange velocity (*w*_t,1_) on NCSD of UFP in the lower canyon at the quasi-steady state, i.e. for Case *w*_t,1_-LL (*w*_t,1_ = 0.012 m s^−1^), Case *w*_t,1_-L (*w*_t,1_ = 0.016 m s^−1^), Case BASE (*w*_t,1_ = 0.02 m s^−1^), Case *w*_t,1_-H (*w*_t,1_ = 0.024 m s^−1^) and Case *w*_t,1_-HH (*w*_t,1_ = 0.028 m s^−1^). The exchange velocity may be significantly influenced by the external wind and turbulence above the canyon, as well as the atmospheric stability (Ramamurthy et al., 2007). The model predicts that the NCSD of UFP is higher when the exchange velocity is lower; this behaviour is especially pronounced for bins near the nucleation-mode peak diameters (in bin 5 with bin bounds of [19.8 nm, 26.9 nm]). The lowest exchange velocity (0.012 m s^−1^) in the Case EX-LL represents the worst ventilation scenarios and the NCSD is highest; Particles are not efficiently ventilated out of the street canyon under lower exchange velocities. This model behaviour is expected: under low wind conditions there will be poor ventilation and particles/pollutants will tend to be trapped in the street canyon; and this effect will be size-dependent because of the different concentration gradients for different particle sizes (cf. the background and box NCSDs in Fig. 4a). Fig. 4 (b) also shows that the number concentrations in the Aitken mode drop more quickly as the exchange velocity increases than the nucleation mode. The number concentration in the accumulation mode decreases modestly with the increase in the exchange velocity. The accumulation mode may be less influenced by evaporation and more influenced by exchange. The effect of evaporation at other modes tends to be more significant.

Fig. 4 (c) shows the percentage of underestimation by the ‘one-box’ model on the number concentration in the lower canyon under a variety of exchange velocities (*w*_t,1_), compared to the two-box model. Underestimation by the ‘one-box’ model decreases slightly with an increase in the exchange velocity, and this effect is more significant for larger diameter particles. The ‘one-box’ model will perform better for scenarios with stronger wind conditions. These size-dependent patterns reflect the characteristics for a single heterogeneity coefficient (i.e. η = 0.5 used for all cases) in Fig. 3 (c). The change of heterogeneity coefficient plays a dominant role in this underestimation by the ‘one-box’ model in such scenarios. Fig. 4 (d) further indicates that the percentage of underestimation by the ‘one-box’ model at the nucleation mode tends to be less influenced by the changes in exchange velocities, but would have more significant effect for the accumulation mode. The accumulation mode in the model is closer to a microphysically-passive scalar and more influenced by the exchange and emissions. On the other hand, the nucleation mode is influenced significantly by evaporation and the slight changes in the underestimation by the ‘one-box’ model in Fig. 4 (d) indicates that the effect of evaporation is more pronounced than the effect of exchange.

### 3.4 Effect of box height ratio

**Fig. 5:**
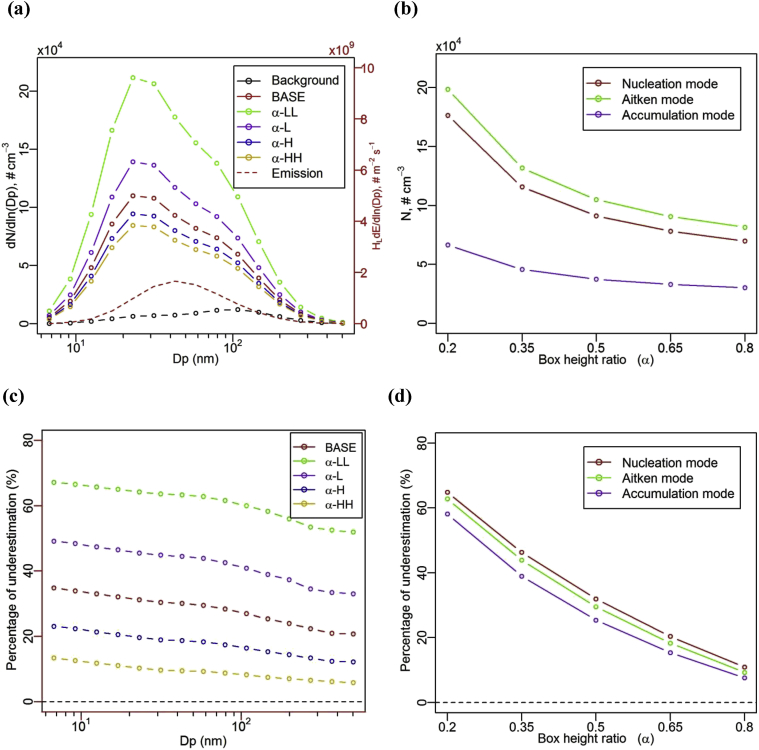
Effect of box height ratio (α) on (a) number concentration size distribution of UFP, (b) number concentrations of UFP for different modes, (c) the percentage of underestimation by the ‘one-box’ model in different size and (d) in different modes, for the lower canyon at the quasi-steady state.

Fig. 5 (a) illustrates the effect of box height ratio (α) on the NCSD of UFP in the lower canyon at the steady state, i.e. for Case α-LL ($w_{t,0}$  = 0.2), Case α-L (α  = 0.35), Case BASE (α  = 0.5), Case α-H (α  = 0.65) and Case α-HH (α  = 0.8). Number concentrations of UFP are significantly influenced by the box height ratio and there are extremely high number concentrations at the Nucleation mode peak diameter (bin 5) for the smaller box height ratios. Fig. 5 (b) shows that the dependence of the number concentrations of UFP on α has the same rank order of modes as in Fig. 3 (b) and Fig. 4. (b), i.e. with the highest number concentrations for the Aitken mode followed by the nucleation mode and the accumulation mode. Number concentrations decrease with increased box height ratios for all modes and those decreases are more pronounced at lower box height ratios than at higher box height ratios.

Fig. 5 (c) shows the percentage of underestimation by the ‘one-box’ model on the number concentration in the lower canyon under a variety of box height ratios (α), compared to the two-box model. As expected, the underestimation by the ‘one-box’ model significantly decreases with the increase in the of box height ratio. For Case α-HH (α  = 0.8), this underestimation is smallest among all the tested cases, ranging from about 15% (bin 1) to about 5% (bin 15). For higher *α* values, the upper box is very thin and could serve as a shear layer at the canyon roof level (in one-box model scenarios). In this sense, the one-box model would be very close to the two-box model in terms of predicting the number concentration in the lower canyon. Fig. 5 (d) further shows that the ‘one-box’ model performs better for higher box height ratios for all modes. This is because, at higher box height ratios, the upper box in the two-box model functions as equivalent to a shear layer in the one-box model and the one-box model tends to be closer to the two-box model. The underestimation ‘error’ is larger for the Aitken mode and nucleation mode.

## 4 Conclusions

A two-box model for a compartmentalised street canyon was coupled with ultrafine particle (UFP) microphysics to examine the number concentration size distribution (NCSD) of UFP at ground-level (where human exposure occurs). The model captures the significant contrasts in UFP number concentrations and a measure of the volatility of the multi-SVOC-particles in the lower and upper parts of a street canyon. At quasi-steady state in the model, the NCSD of UFPs in each street canyon compartment is a balance between the processes of emission (in the lower box only), exchange, and evaporation/condensation of semi-volatiles, none of which is negligible. Modelled ground-level UFP number concentrations vary across nucleation, Aitken, and accumulation particle modes as well-defined monotonic functions of canyon compartmentalisation parameters.

Parameters driving the two-box model account for the position of, and exchange of air between, atmospheric compartments induced by the atmospheric flow acting on details of canyon geometry and by the intensity of traffic-induced turbulence. Previous modelling studies have tended to focus on aspect ratio as the driver for partitioning of the canyon into relatively isolated compartments; we note that our results apply equally to shallower canyons where street trees, street furniture, and architectural features produce compartmentalisation. The dominating processes across the three-dimensional undulating interfaces between the compartments in real-world street canyons are represented by three adjustable parameters: heterogeneity coefficient, box-height ratio, and exchange velocity. These conceptual parameters may be derived empirically from other more complex numerical models or field measurements. A full parametric sensitivity study is beyond the scope of the present work. Assuming that a deep or otherwise compartmentalised canyon is represented by a single well-mixed box leads to underestimation of the number concentrations of UFP by several tens of percent typically (size-dependent). In general, the error due to a single well-mixed box assumption is somewhat larger for the nucleation mode than for the Aitken or Accumulation modes.

Understanding the role of canyon compartmentalisation in determining pollutant concentrations provides the opportunity for new and existing street canyons to be engineered to promote ventilation (e.g. by increasing the local traffic-related turbulence, removing any unnecessary architectural elements/trees interfere with and obstruct the flow, or increasing surface heating), so long as the primary cause of high pollutant concentrations in street canyons remains ground-level traffic. When and if urban pollution sources are predominantly at rooftop level and above (e.g. when and if woodfuel heating becomes prevalent in urban areas), it may be advantageous to promote compartmentalisation of street canyon air to slow pollutant transport to the ground level. The presence of street vegetation can not only provide a sink term to reduce pollutant concentration due to deposition, but also reduce the ventilation conditions and the compartment of the street canyon to increase pollutant concentration by altering the street canyon flow. Santiago et al. (2017) investigated extensively the impact of street vegetation on pollutant concentration, focusing the comparison between the effect of deposition and the effect of ventilation reduction. For scenarios with lower tree canopy and with higher deposition velocity, the effect of deposition would be more significant than the effect of ventilation reduction, which may lead to lower pollutant concentrations within the street canyon. When the tree canopy with high-density leaves is close to the building height, the effect of ventilation reduction would be dominant and cause an increase in the street level pollutants. Higher tree canopy may have more effect on the ventilation for the whole canyon, while the lower tree canopy may have more effect on the local ventilation and hence the heterogeneity. Deposition over vegetation was not considered in this study. Future studies may extend the current two-box street canyon model to investigate extensively the effect of vegetation on pollutant concentrations. The evaluation of the current model may be against future numerical LES-UFP models.

## Appendix A Supplementary data

Data_spl_Profile Data_spl_Profile

Supporting Infomation Supporting Infomation
