# Supplementary material for: Modelling traffic-induced multicomponent ultrafine particles in urban street canyon compartments: Factors that inhibit mixing
Source: Environ Pollut. 2018 Jul;238:186–95. doi: 10.1016/j.envpol.2018.03.002 (PMC5971220; doi:10.1016/j.envpol.2018.03.002)
Supplement: Supporting Infomation [file mmc2.zip › EP_Zhong_et_al_2017_Supporting information_R3_Final.docx]

**SUPPORTING INFORMATION**

Modelling traffic-induced multicomponent ultrafine particles in urban street canyon compartments: factors that inhibit mixing

Jian Zhong1, Irina Nikolova1, Xiaoming Cai1*, A. Rob MacKenzie1,2 and Roy M. Harrison1,3

1. School of Geography, Earth & Environmental Sciences, University of Birmingham, Edgbaston, Birmingham, B15 2TT, UK

2. Birmingham Institute of Forest Research, University of Birmingham, Edgbaston, Birmingham, B15 2TT, UK

3. Department of Environmental Sciences / Center of Excellence in Environmental Studies, King Abdulaziz University, PO Box 80203, Jeddah, 21589, Saudi Arabia

**Corresponding author*. Tel.: (0121) 4145533; Fax: (0121) 4145528.

*Email address*: x.cai@bham.ac.uk (X.-M. Cai).

**Parameters of canyon compartmentalisation**

The exchange velocity in the one-box model, , is defined based on a steady state of the street canyon system with a passive scalar emission. The steady state means that the emission rate, the mean wind speed above the canyon, the flux of the passive scalar out of the canyon (), and the spatial-mean concentrations for the canyon box () and the overlying background () are constant with time. This definition adopts the format of Fick’s law and it gives:

(S)

Likewise, the exchange velocities in the two-box model, and are defined based on the same street canyon system except that two boxes are adopted instead of one. Thus the flux from the lower to the upper box equals that from the upper box to the background:

(S)

where and are the concentrations in the lower and upper boxes, respectively.

We define (S)

which is the ratio of the lower box height to the whole canyon height. For an idealised canyon, is also the percentage of the volume of the lower box. The box height ratio will be determined by the street canyon geometry as well as the flow structure emerging from the interaction with the above-canyon flow. Li et al. (2012) suggested that the flow pattern in a deep street canyon (AR=2) may be significantly influenced by street bottom heating based on their large eddy simulations, i.e. the value of may vary from about 0.4 to 0.9 under neutral, weak heating and strong heating conditions. A pitched roof may induce a relatively smaller size of circulation at the upper canyon (Louka et al., 2000), which would also give a high value of . Secondary smaller eddies near street corners may give a very low value of and particles could be trapped in the lower canyon (with high number concentrations) due to the limited ventilation conditions.

Because the total mass inside the street canyon for the one- and two-box models should be same, the following equation holds:

(S)

A ‘zero background’ for a passive scalar has been assumed in many studies (Murena et al., 2011; Murena, 2012; Zhong et al., 2015), i.e. . Then combining Equations (S1-S2) gives. Zhong et al. (2016) defined the heterogeneity coefficient to represent the spatial variability across the two boxes, i.e.

(S)

where . The case represents two homogenous (well-mixed) boxes, since and for a given (non-zero) box height ratio according to Equation (S4). As *η* approaches 1.0, the concentration difference between the two boxes would become the highest under the constraint of (S5), in which and (i.e. the top of the lower box becomes impermeable). In terms of the stirring and mixing inside street canyons, therefore, α defines a relative position in the canyon at which there is reduced mixing, whilst*η* defines the strength or intensity of the mixing. The details of canyon geometry — street trees, street furniture, architectural features — affect both α and *η*; traffic-induced turbulence affects *η* primarily.

According to Equations (S1-S5), we obtain the following equations (will be taken as an important input parameter):

(S)

(S)

**Table S1. Initial/background vapour concentrations on a urban background site (Harrad et al., 2003; Nikolova et al., 2016) (standard deviations of the measurements are shown in parenthesis) and saturation vapour concentration estimated at temperature of 278.15 K based on EPI suite v4.1 (US EPA, 2017).**

| N-alkanes | Initial/background vapour concentration (in ng m-3) | Saturation vapour pressure at 278.15 K (in Pa) |
| --- | --- | --- |
| C16 | 2.47 (1.46) | 1.31E-01 |
| C17 | 3.67 (2.36) | 5.48E-02 |
| C18 | 3.61 (2.12) | 1.53E-02 |
| C19 | 4.07 (1.96) | 6.51E-03 |
| C20 | 3.13 (1.41) | 2.79E-03 |
| C21 | 2.96 (1.61) | 1.19E-03 |
| C22 | 1.64 (0.86) | 5.42E-04 |
| C23 | 1.49 (1.35) | 2.61E-04 |
| C24 | 0.88 (1.25) | 1.16E-04 |
| C25 | 0.78 (0.69) | 6.30E-05 |
| C26 | 0.74 (0.43) | 3.26E-05 |
| C27 | 0.84 (0.45) | 5.26E-06 |
| C28 | 0.76 (0.48) | 8.56E-06 |
| C29 | 0.79 (0.46) | 5.08E-06 |
| C30 | 0.54 (0.37) | 2.85E-06 |
| C31 | 0.50 (0.32) | 1.65E-06 |
| C32 | 0.28 (0.21) | 9.25E-07 |

**(a) Lower box (2 box model)**


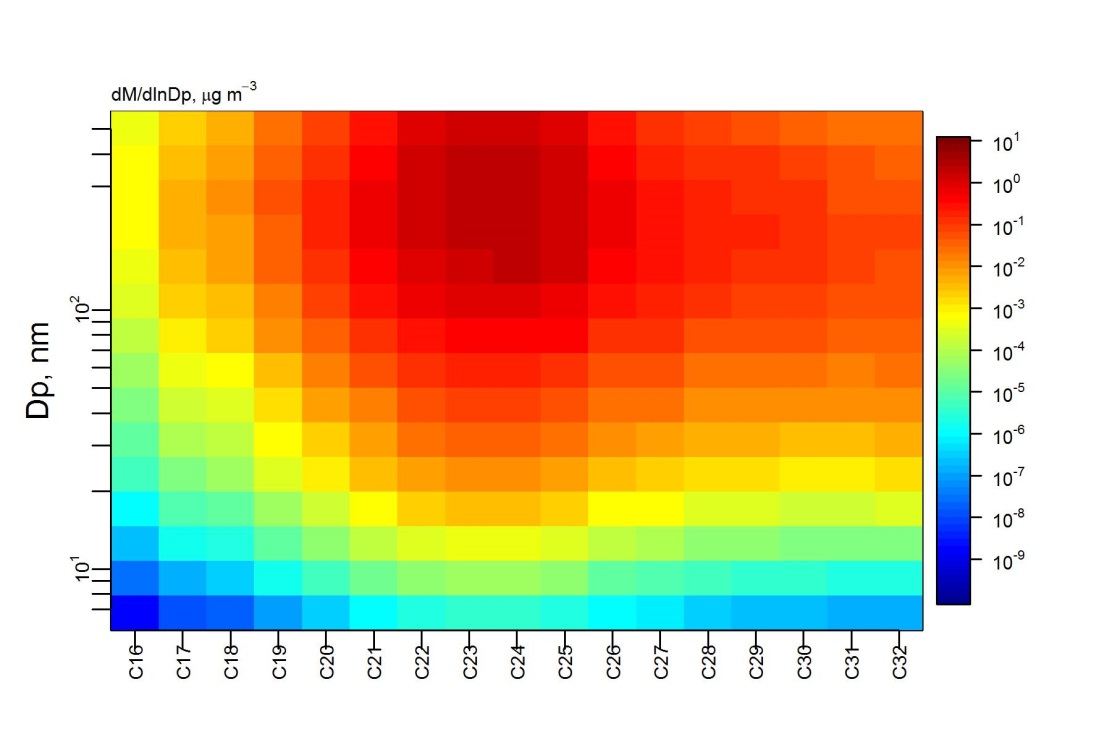


**(b) Upper box (2 box model)**


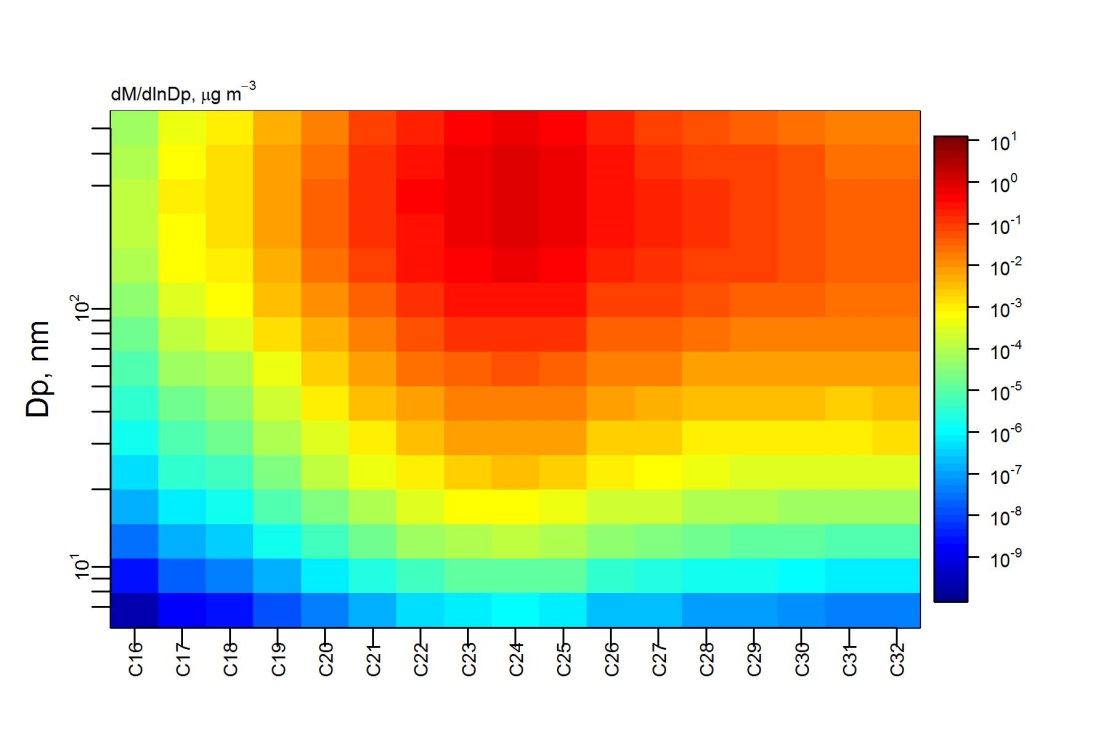


**(c) Single box (1 box model)**


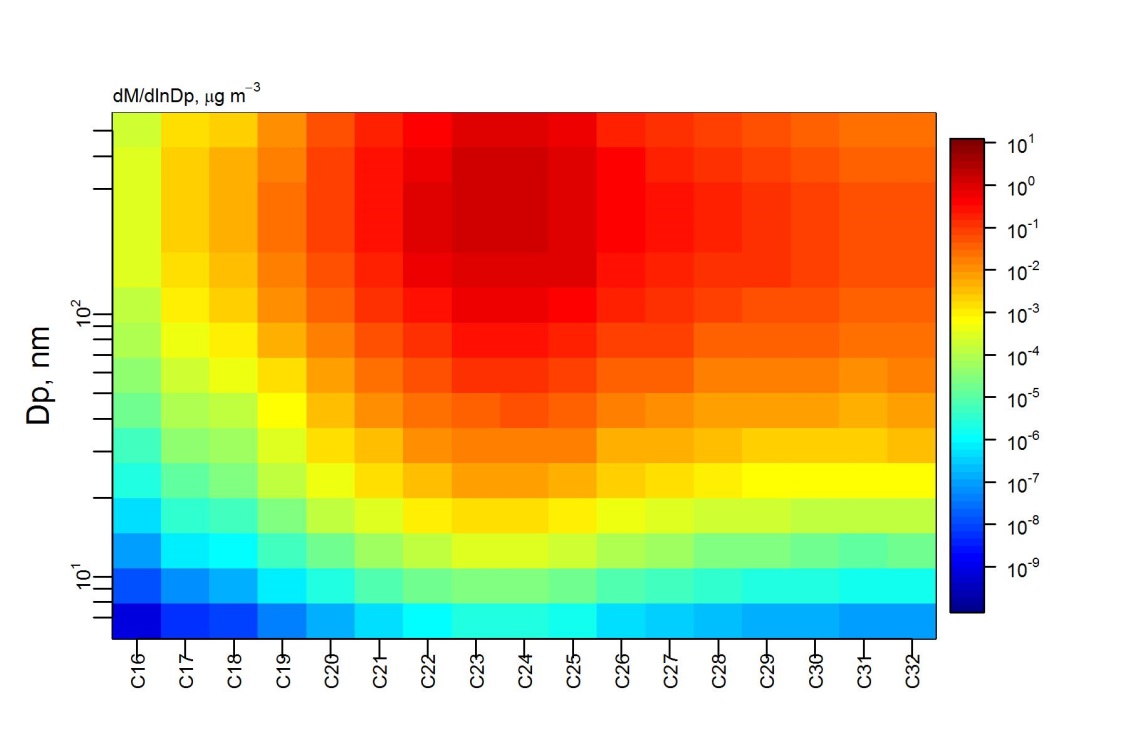


Figure S1. Mulitcomponent mass concentration size distributions, dM/dlnDp (ug m-3), at the quasi-steady state by the 2-box model (a,b) and the 1-box model (c) for Case BASE.

# References:

HARRAD, S., HASSOUN, S., CALLÉN ROMERO, M. A. S. & HARRISON, R. M. 2003. Characterisation and source attribution of the semi-volatile organic content of atmospheric particles and associated vapour phase in Birmingham, UK. *Atmospheric Environment,* 37**,** 4985-4991.

HARRISON, R. M., JONES, A. M., BEDDOWS, D. C. S., DALL'OSTO, M. & NIKOLOVA, I. 2016. Evaporation of traffic-generated nanoparticles during advection from source. *Atmospheric Environment,* 125**,** 1-7.

JACOBSON, M. Z. 2005. *Fundamentals of Atmospheric Modeling,* New York, Cambridge University Press.

JULIN, J., WINKLER, P. M., DONAHUE, N. M., WAGNER, P. E. & RIIPINENT, I. 2014. Near-Unity Mass Accommodation Coefficient of Organic Molecules of Varying Structure. *Environmental Science & Technology,* 48**,** 12083-12089.

LI, X. X., BRITTER, R. E., NORFORD, L. K., KOH, T. Y. & ENTEKHABI, D. 2012. Flow and Pollutant Transport in Urban Street Canyons of Different Aspect Ratios with Ground Heating: Large-Eddy Simulation. *Boundary-Layer Meteorology,* 142**,** 289-304.

LOUKA, P., BELCHER, S. E. & HARRISON, R. G. 2000. Coupling between air flow in streets and the well-developed boundary layer aloft. *Atmospheric Environment,* 34**,** 2613-2621.

MURENA, F. 2012. Monitoring and modelling carbon monoxide concentrations in a deep street canyon: application of a two-box model. *Atmospheric Pollution Research,* 3**,** 311-316.

MURENA, F., DI BENEDETTO, A., D'ONOFRIO, M. & VITIELLO, G. 2011. Mass Transfer Velocity and Momentum Vertical Exchange in Simulated Deep Street Canyons. *Boundary-Layer Meteorology,* 140**,** 125-142.

NIKOLOVA, I., MACKENZIE, A. R., CAI, X. M., ALAM, M. S. & HARRISON, R. M. 2016. Modelling component evaporation and composition change of traffic-induced ultrafine particles during travel from street canyon to urban background. *Faraday Discussions,* 189**,** 529-546.

SANGIORGI, G., FERRERO, L., PERRONE, M. G., PAPA, E. & BOLZACCHINI, E. 2014. Semivolatile PAH and n-alkane gas/particle partitioning using the dual model: up-to-date coefficients and comparison with experimental data. *Environmental Science and Pollution Research,* 21**,** 10163-10173.

SHIN, H. M., MCKONE, T. E., NISHIOKA, M. G., FALLIN, M. D., CROEN, L. A., HERTZ-PICCIOTTO, I., NEWSCHAFFER, C. J. & BENNETT, D. H. 2014. Determining source strength of semivolatile organic compounds using measured concentrations in indoor dust. *Indoor Air,* 24**,** 260-271.

US EPA 2017. Estimation Programs Interface Suite™ for Microsoft® Windows, v 4.11. *United States Environmental Protection Agency***,** Washington, DC, USA.

WEI, W. J., MANDIN, C., BLANCHARD, O., MERCIER, F., PELLETIER, M., LE BOT, B., GLORENNEC, P. & RAMALHO, O. 2016. Distributions of the particle/gas and dust/gas partition coefficients for seventy-two semi-volatile organic compounds in indoor environment. *Chemosphere,* 153**,** 212-219.

ZHONG, J., CAI, X. M. & BLOSS, W. J. 2015. Modelling the dispersion and transport of reactive pollutants in a deep urban street canyon: Using large-eddy simulation. *Environmental Pollution,* 200**,** 42-52.

ZHONG, J., CAI, X. M. & BLOSS, W. J. 2016. Modelling photochemical pollutants in a deep urban street canyon: Application of a coupled two-box model approximation. *Atmospheric Environment,* 143**,** 86-107.
